# Frequency of Toxocariasis among Patients Clinically Suspected to Have Visceral Toxocariasis: A Retrospective Descriptive Study in Sri Lanka

**DOI:** 10.1155/2017/4368659

**Published:** 2017-12-06

**Authors:** Devika Iddawela, Kiruthiha Ehambaram, Dhilma Atapattu, Kalyani Pethiyagoda, Lakmalee Bandara

**Affiliations:** ^1^Department of Parasitology, Faculty of Medicine, University of Peradeniya, Peradeniya, Sri Lanka; ^2^Department of Community Medicine, Faculty of Medicine, University of Peradeniya, Peradeniya, Sri Lanka

## Abstract

**Introduction:**

Human toxocariasis is caused by several species of the nematode* Toxocara*. Two common clinical syndromes are ocular and visceral larva migrans.

**Objectives:**

To determine the* Toxocara* antibody positivity in clinically suspected VLM patients and to describe demographic factors and clinical manifestations of seropositive patients.

**Methods:**

522 clinically suspected patients were studied between 1993 and 2014. Relevant data was gathered from referral letters. Serum samples were subjected to* Toxocara antigen ELISA*.

**Results:**

Overall, seropositivity was 50.2% (262), of which 109 (40.8%) were positive at high level of* Toxocara* antibody carriage and 153 (58.4%) were positive at low levels. The seropositives ranged from 3 months to 70 years (mean = 7.8). Younger age group had higher levels of seropositivity and it was statistically significant. Majority of children under 5 years were seropositive (47.7%, *n* = 125). Seropositivity was common in males (55.3%, *n* = 145). Clinical manifestations of seropositives include lymphadenopathy (24.1%) skin rash (22.5%), dyspnoea (21.7%), fever (21%), hepatosplenomegaly (9.2%), and abdominal pain (3.8%). 197 (75.2%) seropositive cases had eosinophilia. These symptoms were not statistically significant.

**Conclusions:**

This study confirms toxocariasis as an important cause of childhood ill health identifying common clinical symptoms recommending preventive measures to limit transmission.

## 1. Introduction

Toxocariasis, a disease caused by zoonotic roundworms* Toxocara cati* and/or* Toxocara canis* belonging to cats and dogs, respectively, is highly prevalent in many developing countries. Among all different species,* T. canis* is the most important of the parasites causing toxocariasis in humans [1]. Soil contamination with infective eggs is very important in the transmission of toxocariasis to humans [2]. Food borne infection through undercooked infected meats has been documented, although this is a rare route of infection [3, 4]. The risk of acquiring infection is increased with dog ownership, geophagia [5–7], age, geographical location (rural areas), and poor socioeconomic status [8, 9].* Toxocara *spp. cannot develop to the adult worm in humans and remain restricted to the larval form. The migrating larvae cause extensive damage in the organs involved and the condition, characterized by various clinical manifestations, is known as larva migrans.

The clinical spectrum of human toxocariasis ranges from asymptomatic cases to systemic infections. The recognized clinical manifestations include visceral larva migrans (VLM), ocular larva migrans (OLM), covert toxocariasis, and asymptomatic toxocariasis [10]. The clinical syndrome of VLM may be acute or subacute, with hepatomegaly, splenomegaly, pyrexia, gastrointestinal symptoms, cutaneous manifestations, pulmonary involvement, central nervous system involvement, and eosinophilia [11]. Clinical features associated with toxocariasis are common and nonspecific and majority of clinical features present as symptoms. Therefore diagnosis based on clinical findings is unreliable since the* Toxocara* larvae fail to complete their migratory cycle in the human; eggs are not passed in the stool. Definitive diagnosis of toxocariasis is by histological examination for* Toxocara* larvae in biopsy materials. But it is all most impossible to detect larvae in tissues because parasites may be few in the tissues of those infected and unless situated in an organ such as eye may be difficult or impossible to locate. Thus the confirmatory diagnosis of toxocariasis depends greatly on immunological tests.

The seroprevalence rate in the world varies from less than 10% [12, 13] to more than 80% in certain developing countries [14, 15].* Toxocara *infection is more prevalent in less developed tropical countries [11, 16]. The first seroepidemiological study on toxocariasis in Sri Lankan population which was conducted in 2003 on 1020 children in the age group 1–12 years revealed 43% of seropositivity indicating high rate of transmission [7]. There is scarcity of studies done to determine the* Toxocara *antibodies among clinically suspected patients. Therefore the current study was carried out to determine the seroprevalence of* Toxocara* antibodies among suspected VLM patients and to describe demographic factors and clinical manifestations of the seropositive patients.

## 2. Methods

### 2.1. Study Setting and Population

A retrospective study was carried out on all the clinical samples that were referred to the Department of Parasitology, Faculty of Medicine, University of Peradeniya, for serological diagnosis to confirm the etiological diagnosis of clinically suspected VLM patients by consultant physicians and consultant paediatricians from several teaching hospitals, general hospitals, and base hospital in Sri Lanka. All the data (age, sex, presenting complain, and other investigation results) included in the referral letters were recorded and entered into a clinical data storage computer in our department. All the patient files between the years 1995 to 2014 were retrieved and the presenting complaints of all the patients were studied and categorized. Relevant clinical and laboratory data were extracted and entered in a Microsoft excel sheet. Variables included demographic data,* Toxocara* antibody optical density (OD) result, and the various clinical symptoms.

This study has been exempted from ethical clearance by the Ethics Review Committee of the Faculty of Medicine, University of Peradeniya.

### 2.2. Detection of* Toxocara* IgG Antibodies

From 1995 to 2014 April all the* Toxocara* VLM suspect cases were diagnosed via In-House TES-ELISA developed and validated in our laboratory [17]. The microtitration plates were coated with 0.846 *μ*g/ml* Toxocara* excretory secretory antigens and incubated at 4°C overnight. Plates were then washed in washing buffer (PBS with 0.05% Tween 20), postcoated with 100 *μ*l PBS containing 1% bovine serum albumin and 2.5% sucrose, and incubated at room temperature (RT) for one hour. The plates were washed five times with wash buffer. Subsequently 100 *μ*l of diluted (1 : 100) serum samples was added to the test well in duplicate and incubated for one hour at RT. Known negative and positive sera were used as control in each plate. Following the incubation, plates were washed three times in washing buffer to remove unbound serum and 100 *μ*l of horse radish peroxidase conjugated anti-human IgG (Sigma Chem Co.) at a dilution of 1 : 5000 was added to each well. Plates were incubated for 1 hour at RT. Subsequently 100 *μ*l of the substrate o-phenylenediamine dihydrochloride and 2 mg tablets in 3% hydrogen peroxide solution (Sigma-Aldrich, India) were added to each well. After incubating for 20 min at RT, 100 *μ*l of 3 M H_2_SO_4_ was added to each to stop the reaction. The optical density (OD) at 492 nm was measured with an automated ELISA reader. An OD value less than or equal to 0.2 was considered as negative and that greater than or equal to 0.7 was considered positive. While the values within this range (0.2 < OD < 0.7) were considered as a light infection or past infection, the OD value > 0.7 was considered as high positive indicating recent infection [17].

### 2.3. Statistical Analysis

Age and gender distribution of the population was described using parametric methods. Following this, gender and age categories and serological status classified as negative, low positive, and high positive were subjected to nonparametric methods. Additionally one-way ANOVA was applied to serology categories and mean age to confirm above results. Further analysis was carried out amalgamating low positive and high positives as one group.

Finally prevalence of clinical symptoms such as eosinophilia, cervical lymphadenopathy, generalized lymphadenopathy, presence of rash, hepatomegaly, splenomegaly, hepatosplenomegaly, fever, lower and upper respiratory tract symptoms, gastrointestinal symptoms, myalgia, arthralgia, pallor, thrombocytopenia, fits, and ecchymosis was cross tabbed with serological status and chi square test applied to each of these and tested whether these were significantly associated.

*P* value of less than 0.05 was considered to indicate statistical significance.

## 3. Results

The study population is comprised of a total of 511 study subjects of which 312 (61.1%) were males and 199 (38.9%) were females (Table 1). Mean age of males was 6.5 years (±7.9) and for females it was 9.5 years (±12.9). Females were found to be significantly older than males (*F* = 10.88, df = 1, *P* < 0.001).

Of the total of 511 clinically suspected patients, 259 (50.68%) were positive for presence of anti TES antibodies. Of the 259 seropositive cases 40.9.% (*n* = 106) were positive at high level of* Toxocara *antibody carriage indicating recent infection (OD ≥ 0.7). The OD values ranged from 0.7 to 2.708.

Low level of* Toxocara *antibody carriage (OD 0.2 to 0.69) indicating light infection or past exposure was observed in 59.07% (*n* = 153) of the seropositive population.

A total of 49.4% of males and 52.8% of females were found to be serologically positive. There was no statistically significant association with gender and serological positivity (Table 2).

Higher levels of seropositivity was found among the younger age groups (<10 years) and it was found that age was significantly associated with seropositivity (Tables 3 and 4).

Of a number of relevant clinical features that was prevalent, the prevalence of eosinophilia was highest (72.6%) among the study population, followed by fever (23.3%), lower respiratory tract symptoms (20.4%), and ecchymosis (20.3%). However, when considering statistical associations with serological status, generalized lymphadenopathy showed a highly significant association (*P* < 0.004) with seropositivity followed by cervical lymphadenopathy (*P* < 0.032) (Table 5).

## 4. Discussion

Toxocariasis represents one of the most common parasitic zoonotic infections worldwide particularly in developing countries and some tropical islands [18]. Sri Lanka being a tropical island and one of the developing countries has been a common site for* Toxocara *infections. In this study, a positive antibody carriage of 50.68% was observed among the clinically suspected patients indicating this infection is common in Sri Lanka. This level of antibody carriage is higher than the 43% of seropositivity reported in a seroepidemiological study carried out in children aged between 1 and 12 year in Sri Lanka [7]. But this is much lower than the 86% reported in a rural Colombian study where the study group of 82 children was from a very low socioeconomic level [1]. In 41.6% of the study population a high level of seropositivity was observed implicating recent or heavy infection. A community based seroepidemiological study carried out in children aged 1−12 years of a rural area in Sri Lanka reported much lower rate (16.6%) of children having recent infection [7]. Similarly a community based Venesulan study considering the seropositivity at the high cut-off titre (similar to value used in this study) showed 20% positivity for urban slum dwellers and 25.6% for the rural farming community for all ages. In our study we included only symptomatic patients. This could be the reason for high rate of recent infection reported in our study.

The age of a typical VLM patient is a child between the ages of 2 and 7 years [1]. Among all the seropositive cases (*n* = 262) we have identified a large proportion of children aged <10 years and the age was significantly associated with seropositivity. This was similar to another study conducted only on children which had a mean age of 7.3 years [19]. In our study, active or recent infection was common among younger children (mean age 6.15 years) compared to those (mean age 9.2 years) with past or mild infection. When Beaver first identified* Toxocara *larvae as the aetiological agent for VLM, he postulated that zoonotic toxocariasis was probably common, especially among children [20]. All subsequent studies support Beaver's observation. Although in most populations studied young children have exhibited the highest prevalence [21–23] some studies have failed to show the age-related correlation [1, 24, 25]. Toxocariasis predominantly affects the children due to their close contact with pet animals (dogs and cats), geophagia, poor hygienic practices, and playing in contaminated sand pits. Present study was unable to demonstrate any significant association between sex and seropositivity. This is in agreement with the studies of Thomson et al. (1986); Cortés et al. (2015) [18, 25]. In contrary to our study findings Brazil [26] and in India [27] have reported that male children were more prone to infection.

The most common symptoms which lead to a consultation with the doctor were fever, lower respiratory tract difficulties, and enlarged lymph nodes. The physician suspected a parasitic infection mainly due to eosinophilia and/or lymphadenopathy. Eosinophilia was the predominant clinical feature (72.19%) and main predictor for clinical suspicion of toxocariasis among the study population. A prior study stated eosinophilia as a reason for performing* Toxocara* diagnosis [27]. Similar to our study, Fernando et al. in 2007 [28] documented a high rate of eosinophilia among seropositive paediatric population. However, there was no statistically significant association between eosinophilia and* Toxocara* seropositivity in our study. In conformity to our study, studies in Iran and in Brazil did not documented a statistically significant correlation between eosinophilia and* Toxocara* seropositivity [29, 30]. Roldán et al. in 2008 [11] have documented a statistically significant association between* Toxocara *positive serology and eosinophilia in preschool children. Although the exact role of eosinophil cells in fighting against helminth infections is not understood, Hõrak et al. (2006) stated that the parasite manipulates the host cells to produce increased number of eosinophils thereby blocking the development of inflammatory responses [31].

In our study, there was a statistically significant association between lymphadenopathy (cervical and generalized) and seropositivity. Similarly, several studies have identified lymphadenopathy as a most frequently encountered clinical feature among* Toxocara *infected children [32]. Of the total seropositive population, 22.5% had skin rash and difficulty in breathing was a common presenting symptom (21.4%) among seropositive study group. Some studies documented that* T. canis* infection leads to allergic sensitization [33]. Diverse manifestations including wheezing and skin ecchymosis have been attributed to toxocariasis in children in hospital-based studies in Sri Lanka [33, 34]. Studies have shown the capability of* T. canis* to increase the vulnerability to episodic wheezing in some patients due to activation of host defense mechanism to the parasite or due to Th2-type reaction to inhaled allergens, set up by* T. canis *[35, 36]. Several studies have proved positive correlation between asthma and* Toxocara* infection particularly in children [3, 37]. Another prominent symptom was fever (20.9%) which is similar to a study conducted in 2001 [35]. A review report on toxocariasis in Japan and several other case reports also concluded that fever is a prominent symptom in patients with toxocariasis regardless of age group [38]. A community based study conducted in children in a rural area of Sri Lanka showed a significant association between fever and* Toxocara *seropositivity [7]. The other common symptom mentioned in several reports is hepatosplenomegaly which make the diagnosis of toxocariasis more likely when accompanied by history of pica and involvement of multiple systems [10, 36]. However, our study observed only 6.17% of patients with hepatosplenomegaly having seropositivity.

Number of prior studies state that muscular pain is observed in toxocariasis infection [27, 39]. Although we observed several patients with myalgia the incidence was calculated up to only 4.2%. Arthralgia was observed in a similar percentage of seropositive patients (4.2%) which differs largely compared to another study that reports an incidence of 19.6% [34].

In conclusion this study reported a* Toxocara* aetiology in considerably large proportion of symptomatic patients. Among the suspected VLM patients, present study identifies eosinophilia, lymphadenopathy, fever, and upper respiratory tract difficulties more frequently in the seropositive group compared to the rest of the symptoms indicating that toxocariasis has caused considerable childhood morbidity in this study group and confirms the need to recognize toxocariasis as a disease entity emphasizing urgent need for implementing preventive measures. The limitation of the study is that this study is a retrospective descriptive study; therefore we were unable to follow-up these patients for clinical and serological cure. Further studies are recommended to research into these areas in children.

## Figures and Tables

**Table 1:** Description of the study population by age and gender.

Age (years)	Males *n* (%)	Females *n* (%)
0 to 4.9	143 (45.8)	76 (38.2)
5 to 9.9	125 (40.1)	73 (36.7)
10 to 14.9	33 (10.6)	29 (14.6)
15 to 19.9	1 (0.3)	2 (1.0)
≥20	10 (3.2)	19 (9.5)

Total	312 (100)	199 (100)

**Table 2:** Description of the study population by serology and gender.

Serology	Males *n* (%)	Females *n* (%)
Negative	158 (50.6)	94 (47.2)
Low positive	88 (28.2)	65 (32.7)
High positive	66 (21.2)	40 (20.1)

Total	312 (100)	199 (100)

*χ* = 1.16; df = 2; *P* = 0.56.

**Table 3:** Description of the study population by serology and age groups.

Age (years)	Negative *n* (%)	Low positive *n* (%)	High positive *n* (%)
0 to 4.9	117 (46.4)	54 (35.3)	48 (45.3)
5 to 9.9	90 (35.7)	65 (42.5)	43 (40.6)
10 to 14.9	27 (10.7)	21 (13.7)	14 (13.2)
15 to 19.9	2 (0.8)	1 (0.7)	0 (0)
≥20	16 (6.3)	12 (7.8)	1 (0.9)

Total	252 (100)	153 (100)	106 (100)

**Table 4:** Description of the study population by serology and mean age.

Serology	Mean age (years)	Range (years)	95% CI
Negative	7.32	0.25 to 63	6.13 to 8.50
Low positive	9.22	0.30 to 70	7.13 to 11.32
High positive	6.15	0.50 to 65	4.89 to 7.41

Total	7.64	0.25 to 70	6.75 to 8.54

*F* = 3.06; df = 2; *P* = 0.048.

**Table 5:** Prevalence of clinical features and association with serological status.

Serology	Negative *n*	Low positive *n*	High positive *n*	Total number of seropositives *N*/%	Number of cases within total study population (%)	Chi-value	Significance (*P*)
Eosinophilia	174	116	81	197 (76.06)	371 (72.6)	3.17	0.205
Cervical lymphadenopathy	25	24	21	45 (17.37)	70 (13.7)	6.904	0.032^*∗*^
Generalized lymphadenopathy	41	10	8	18 (6.9)	59 (11.5)	10.926	0.004^*∗∗*^
Rash	17	7	5	12 (4.6)	29 (5.7)	1.067	0.586
Hepatomegaly	10	2	4	6 (2.3)	16 (3.1)	—	—
Splenomegaly	2	1	0	1	3 (0.6)	—	—
Hepatosplenomegaly	23	8	8	16 (6.17)	39 (7.6)	2.054	0.358
Fever	66	35	18	53 (20.46)	119 (23.3)	3.563	0.168
Difficulty in breathing	50	29	25	54 (20.84)	104 (20.4)	0.908	0.635
Cough	4	4	1	5 (1.9)	9 (1.8)	—	—
Abdominal pain	7	4	4	8 (3)	15 (2.9)	—	—
Myalgia	6	5	5	10 (3.8)	16 (3.1)	—	—
Arthralgia	11	10	1	11 (4.2)	22 (4.3)	—	—
Pallor	4	2	2	4 (1.5)	8 (1.6)	—	—
Thrombocytopenia	9	6	4	10 (3.8)	19 (3.7)	—	—
Fits	2	1	1	2	4 (0.8)	—	—
Ecchymosis	53	33	17	50 (19.3)	103 (20.3)	1.427	0.490

^*∗*, *∗∗*^ *P* value < 0.05.
